# Crystal structure of 2-cyano-1-methyl­pyridinium perchlorate

**DOI:** 10.1107/S2056989015019155

**Published:** 2015-10-17

**Authors:** Vu D. Nguyen, Cameron A. McCormick, Joel T. Mague, Lynn V. Koplitz

**Affiliations:** a Department of Chemistry, Loyola University, New Orleans, LA 70118, USA; b Department of Chemistry, Tulane University, New Orleans, LA 70118, USA

**Keywords:** crystal structure, salt, pyridinium, perchlorate, hydrogen bonding

## Abstract

The asymmetric unit of the title salt, C_7_H_7_N_2_^+^·ClO_4_^−^, comprises two independent formula units. The solid-state structure comprises corrugated layers of cations and of anions, approximately parallel to (010). The supra­molecular layers are stabilized and connected by C—H⋯O hydrogen bonding to consolidate a three-dimensional architecture. A close pyridin­ium–perchlorate N⋯O contact [2.867 (5) Å] is noted. The crystal was refined as an inversion twin.

## Related literature   

For structures of other salts of the 2-cyano-1-methyl­pyridinium cation, see: Koplitz *et al.* (2012 ▸); Kammer *et al.* (2013 ▸); Vaccaro *et al.* (2015 ▸). For structures of salts of the isomeric 2-cyano­anilinium cation, see: Zhang (2009 ▸); Cui & Chen (2010 ▸).
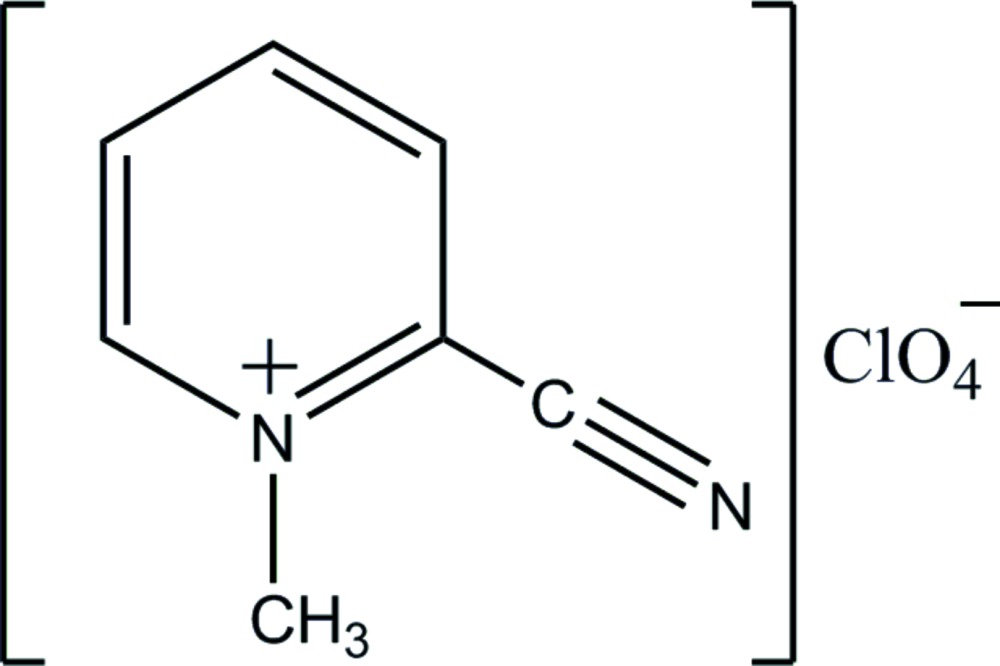


## Experimental   

### Crystal data  

C_7_H_7_N_2_^+^·ClO_4_^−^
*M* *_r_* = 218.60
Monoclinic, 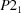

*a* = 8.0112 (12) Å
*b* = 7.7011 (12) Å
*c* = 14.742 (2) Å
β = 90.982 (2)°
*V* = 909.4 (2) Å^3^
*Z* = 4
Mo *K*α radiation
μ = 0.41 mm^−1^
*T* = 150 K
0.19 × 0.14 × 0.13 mm

### Data collection  

Bruker SMART APEX CCD diffractometer
Absorption correction: multi-scan (*TWINABS*; Sheldrick, 2009 ▸) *T*_min_ = 0.93, *T*_max_ = 0.95
22843 measured reflections
22843 independent reflections
20913 reflections with *I* > 2σ(*I*)
*R*_int_ = 0.051

### Refinement  

*R*[*F* ^2^ > 2σ(*F* ^2^)] = 0.043
*wR*(*F* ^2^) = 0.109
*S* = 1.00
22843 reflections
256 parameters
1 restraint
H-atom parameters constrained
Δρ_max_ = 0.30 e Å^−3^
Δρ_min_ = −0.34 e Å^−3^
Absolute structure: Flack *x* determined using 1908 quotients [(*I* ^+^)−(*I* ^−^)]/[(*I* ^+^)+(*I* ^−^)] (Parsons *et al.*, 2013 ▸)
Absolute structure parameter: 0.04 (3)

### 

Data collection: *APEX2* (Bruker, 2014 ▸); cell refinement: *SAINT* (Bruker, 2014 ▸); data reduction: *SAINT* and *CELL_NOW* (Sheldrick, 2008*a* ▸); program(s) used to solve structure: *SHELXT* (Sheldrick, 2015*a* ▸); program(s) used to refine structure: *SHELXL2014* (Sheldrick, 2015*b* ▸); molecular graphics: *DIAMOND* (Brandenburg & Putz, 2012 ▸); software used to prepare material for publication: *SHELXTL* (Sheldrick, 2008*b* ▸).

## Supplementary Material

Crystal structure: contains datablock(s) global, I. DOI: 10.1107/S2056989015019155/tk5395sup1.cif

Structure factors: contains datablock(s) I. DOI: 10.1107/S2056989015019155/tk5395Isup2.hkl

Supporting information file. DOI: 10.1107/S2056989015019155/tk5395Isup3.cml

**Fig. 1.**
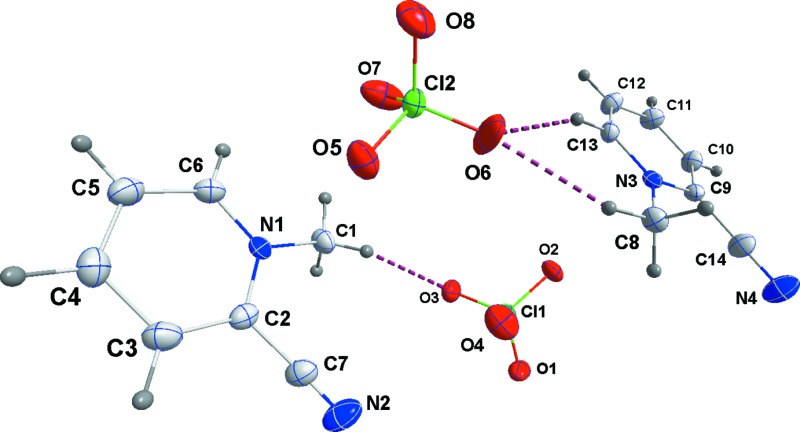
Perspective view of the asymmetric unit with 50% probability ellipsoids. C—H···O interactions are shown by dotted lines.

**Fig. 2.**
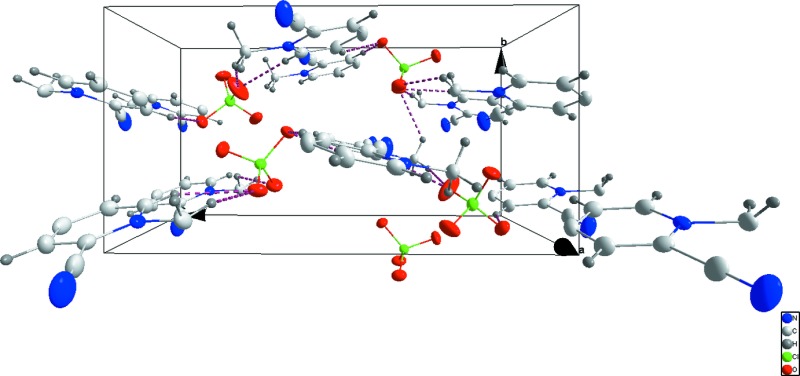
Packing viewed down the a axis showing an edge view of two corrugated layers and the C—H···O interaction (dotted line) holding them together.

**Fig. 3.**
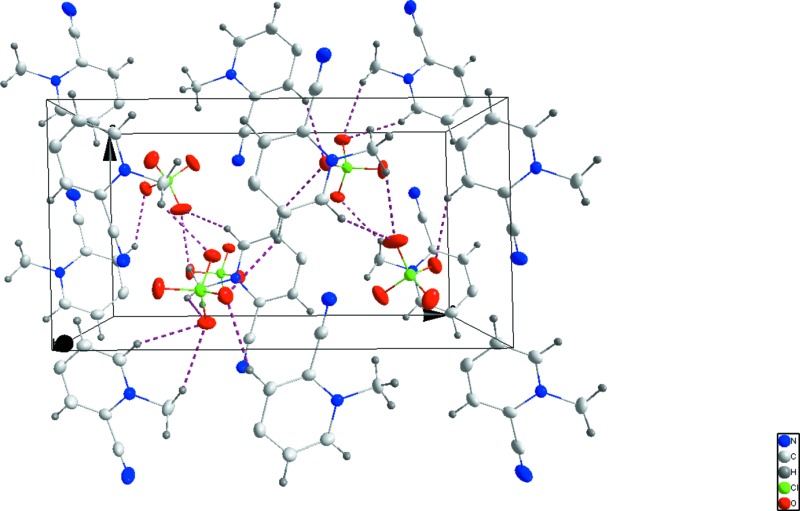
Packing viewed down the b axis providing a plan view of the corrugated sheets with C—H···O interactions shown as dotted lines.

. DOI: 10.1107/S2056989015019155/tk5395fig1.tif
Perspective view of the asymmetric unit with 50% probability ellipsoids. C—H⋯O inter­actions are shown by dotted lines.

a . DOI: 10.1107/S2056989015019155/tk5395fig2.tif
Packing viewed down the *a* axis showing an edge view of two corrugated layers and the C—H⋯O inter­action (dotted line) holding them together.

b . DOI: 10.1107/S2056989015019155/tk5395fig3.tif
Packing viewed down the *b* axis providing a plan view of the corrugated sheets with C—H⋯O inter­actions shown as dotted lines.

CCDC reference: 1430590

Additional supporting information:  crystallographic information; 3D view; checkCIF report

## Figures and Tables

**Table 1:** Hydrogen-bond geometry (, )

*D*H*A*	*D*H	H*A*	*D* *A*	*D*H*A*
C1H1*A*O1^i^	0.98	2.53	3.441(5)	154
C1H1*C*O3	0.98	2.52	3.164(5)	123
C3H3O5^ii^	0.95	2.54	3.326(5)	140
C5H5O8^iii^	0.95	2.66	3.262(6)	122
C6H6O1^i^	0.95	2.55	3.415(6)	152
C6H6O4^i^	0.95	2.65	3.534(6)	155
C8H8*A*O1^iv^	0.98	2.55	3.294(6)	132
C8H8*B*O7^v^	0.98	2.57	3.538(6)	169
C8H8*C*O6	0.98	2.51	3.425(5)	156
C10H10O2^vi^	0.95	2.51	3.367(5)	150
C12H12O2^vii^	0.95	2.52	3.347(5)	145
C13H13O6	0.95	2.35	3.247(6)	156

Symmetry codes: (i) 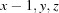; (ii) 
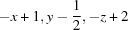
; (iii) 
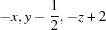
; (iv) 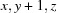; (v) 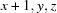; (vi) 
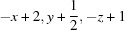
; (vii) 
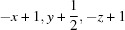
.

## supplementary crystallographic information

### S1. Comment

The asymmetric unit comprises two independent formula units. A portion of the C—H···O hydrogen bonding network which aids the packing of the several ions is shown in Fig. 1 with a fuller depiction appearing in Figs 2 and 3. The solid state structure consists of corrugated layers of cations and anions formed by C—H···O hydrogen bonding between them and approximately parallel to (010). These layers are held to one another by additional C—H···O interactions. The overall structure is essentially the same as found for the tetrafluoroborate salt (Vaccaro *et al.*, 2015).

### S2. Experimental

2-Cyano-1-methylpyridinium iodide (0.42 g, 1.70 mmol; m.p. 146–150°) was dissolved in a solution of silver perchlorate previously prepared by reacting Ag_2_O (0.20 g, 0.86 mmol) with 1*M* aqueous HClO_4_ (1.8 ml) in 8.0 ml of H_2_O. After stirring, the precipitated AgI was removed by vacuum filtration. The filtrate was slowly evaporated to dryness in a freezer at about -5° over several months to form crystals suitable for single-crystal X-ray diffraction.

### S3. Refinement

H-atoms attached to carbon were placed in calculated positions (C—H = 0.95 - 0.98 Å). All were included as riding contributions with isotropic displacement parameters 1.2 - 1.5 times those of the attached atoms. The crystal was refined as a 2-component twin.

### Crystal data

**Table:**

C_7_H_7_N_2_^+^·ClO_4_^−^	*F*(000) = 448
*M**_r_* = 218.60	*D*_x_ = 1.597 Mg m^−^^3^
Monoclinic, *P*2_1_	Mo *K*α radiation, λ = 0.71073 Å
*a* = 8.0112 (12) Å	Cell parameters from 9987 reflections
*b* = 7.7011 (12) Å	θ = 2.5–29.2°
*c* = 14.742 (2) Å	µ = 0.41 mm^−^^1^
β = 90.982 (2)°	*T* = 150 K
*V* = 909.4 (2) Å^3^	Block, colourless
*Z* = 4	0.19 × 0.14 × 0.13 mm

### Data collection

**Table:**

Bruker SMART APEX CCD diffractometer	22843 independent reflections
Radiation source: fine-focus sealed tube	20913 reflections with *I* > 2σ(*I*)
Graphite monochromator	*R*_int_ = 0.051
Detector resolution: 8.3660 pixels mm^-1^	θ_max_ = 29.2°, θ_min_ = 2.5°
φ and ω scans	*h* = −10→10
Absorption correction: multi-scan (*TWINABS*; Sheldrick, 2009)	*k* = −10→10
*T*_min_ = 0.93, *T*_max_ = 0.95	*l* = −20→20
22843 measured reflections	

### Refinement

**Table:**

Refinement on *F*^2^	Secondary atom site location: difference Fourier map
Least-squares matrix: full	Hydrogen site location: inferred from neighbouring sites
*R*[*F*^2^ > 2σ(*F*^2^)] = 0.043	H-atom parameters constrained
*wR*(*F*^2^) = 0.109	*w* = 1/[σ^2^(*F*_o_^2^) + (0.0531*P*)^2^] where *P* = (*F*_o_^2^ + 2*F*_c_^2^)/3
*S* = 1.00	(Δ/σ)_max_ < 0.001
22843 reflections	Δρ_max_ = 0.30 e Å^−^^3^
256 parameters	Δρ_min_ = −0.34 e Å^−^^3^
1 restraint	Absolute structure: Flack *x* determined using 1908 quotients [(*I*^+^)-(*I*^-^)]/[(*I*^+^)+(*I*^-^)] (Parsons *et al.*, 2013)
Primary atom site location: structure-invariant direct methods	Absolute structure parameter: 0.04 (3)

### Special details

**Table:**

Experimental. The diffraction data were obtained from 3 sets of 400 frames, each of width 0.5° in ω, collected at φ = 0.00, 90.00 and 180.00° and 2 sets of 800 frames, each of width 0.45° in φ, collected at ω = -30.00 and 210.00°. The scan time was 15 sec/frame. Analysis of 3152 reflections having I/σ(I) > 13 and chosen from the full data set with *CELL_NOW* (Sheldrick, 2008a) showed the crystal to belong to the monoclinic system and to be twinned by a 180° rotation about c*. The raw data were processed using the multi-component version of *SAINT* under control of the two-component orientation file generated by *CELL_NOW*.
Geometry. All e.s.d.'s (except the e.s.d. in the dihedral angle between two l.s. planes) are estimated using the full covariance matrix. The cell e.s.d.'s are taken into account individually in the estimation of e.s.d.'s in distances, angles and torsion angles; correlations between e.s.d.'s in cell parameters are only used when they are defined by crystal symmetry. An approximate (isotropic) treatment of cell e.s.d.'s is used for estimating e.s.d.'s involving l.s. planes.
Refinement. Refinement of *F*^2^ against ALL reflections. The weighted *R*-factor *wR* and goodness of fit *S* are based on *F*^2^, conventional *R*-factors *R* are based on *F*, with *F* set to zero for negative *F*^2^. The threshold expression of *F*^2^ > σ(*F*^2^) is used only for calculating *R*-factors(gt) *etc*. and is not relevant to the choice of reflections for refinement. *R*-factors based on *F*^2^ are statistically about twice as large as those based on *F*, and *R*- factors based on ALL data will be even larger. H-atoms attached to carbon were placed in calculated positions (C—H = 0.95 - 0.98 Å). All were included as riding contributions with isotropic displacement parameters 1.2 - 1.5 times those of the attached atoms. Refined as a 2-component twin.

### Fractional atomic coordinates and isotropic or equivalent isotropic displacement parameters (Å^2^)

**Table:**

	*x*	*y*	*z*	*U*_iso_*/*U*_eq_	
N1	0.2547 (4)	0.1723 (5)	0.8742 (2)	0.0186 (7)	
N2	0.6652 (5)	0.0531 (8)	0.8975 (3)	0.0424 (14)	
C1	0.3149 (5)	0.1871 (7)	0.7797 (3)	0.0268 (10)	
H1A	0.2255	0.2339	0.7406	0.040*	
H1B	0.3474	0.0721	0.7577	0.040*	
H1C	0.4115	0.2651	0.7785	0.040*	
C2	0.3617 (5)	0.1208 (6)	0.9418 (3)	0.0204 (10)	
C3	0.3089 (5)	0.1017 (7)	1.0292 (3)	0.0262 (11)	
H3	0.3845	0.0658	1.0759	0.031*	
C4	0.1425 (5)	0.1359 (7)	1.0482 (3)	0.0261 (11)	
H4	0.1033	0.1237	1.1083	0.031*	
C5	0.0358 (5)	0.1874 (7)	0.9798 (3)	0.0265 (11)	
H5	−0.0780	0.2106	0.9921	0.032*	
C6	0.0947 (5)	0.2053 (7)	0.8927 (3)	0.0237 (10)	
H6	0.0208	0.2414	0.8453	0.028*	
C7	0.5316 (5)	0.0839 (8)	0.9162 (3)	0.0266 (11)	
Cl1	0.77813 (11)	0.34996 (12)	0.68489 (7)	0.0185 (2)	
O1	0.9290 (4)	0.2488 (5)	0.6789 (3)	0.0300 (8)	
O2	0.7857 (4)	0.4925 (4)	0.6214 (2)	0.0263 (7)	
O3	0.6375 (4)	0.2420 (5)	0.6622 (3)	0.0295 (9)	
O4	0.7628 (4)	0.4142 (5)	0.7754 (2)	0.0370 (9)	
N3	0.7638 (4)	0.8780 (5)	0.6208 (3)	0.0167 (8)	
N4	1.1905 (4)	0.9228 (7)	0.6055 (3)	0.0374 (12)	
C8	0.8152 (5)	0.8446 (7)	0.7165 (3)	0.0214 (9)	
H8A	0.8523	0.9534	0.7448	0.032*	
H8B	0.9070	0.7604	0.7181	0.032*	
H8C	0.7203	0.7979	0.7497	0.032*	
C9	0.8788 (4)	0.9197 (6)	0.5573 (3)	0.0187 (9)	
C10	0.8332 (5)	0.9575 (7)	0.4697 (3)	0.0232 (10)	
H10	0.9152	0.9855	0.4263	0.028*	
C11	0.6647 (5)	0.9542 (7)	0.4452 (3)	0.0254 (10)	
H11	0.6295	0.9816	0.3850	0.030*	
C12	0.5499 (5)	0.9103 (7)	0.5099 (4)	0.0253 (11)	
H12	0.4345	0.9046	0.4942	0.030*	
C13	0.6021 (4)	0.8747 (6)	0.5969 (3)	0.0214 (10)	
H13	0.5216	0.8472	0.6413	0.026*	
C14	1.0526 (5)	0.9217 (7)	0.5867 (4)	0.0259 (11)	
Cl2	0.26845 (11)	0.66595 (14)	0.81449 (7)	0.0213 (2)	
O5	0.3040 (4)	0.5521 (5)	0.8898 (2)	0.0329 (9)	
O6	0.4209 (4)	0.7202 (7)	0.7750 (3)	0.0629 (16)	
O7	0.1683 (5)	0.5742 (6)	0.7490 (3)	0.0436 (10)	
O8	0.1756 (4)	0.8134 (5)	0.8449 (3)	0.0405 (10)	

### Atomic displacement parameters (Å^2^)

**Table:**

	*U*^11^	*U*^22^	*U*^33^	*U*^12^	*U*^13^	*U*^23^
N1	0.0224 (14)	0.0147 (18)	0.0187 (19)	−0.0010 (15)	0.0002 (13)	0.0016 (17)
N2	0.0285 (19)	0.069 (4)	0.030 (3)	0.009 (2)	−0.0004 (17)	−0.009 (3)
C1	0.034 (2)	0.026 (3)	0.020 (2)	0.004 (2)	0.0063 (17)	0.004 (2)
C2	0.0181 (16)	0.018 (3)	0.025 (3)	−0.0009 (15)	−0.0016 (15)	−0.003 (2)
C3	0.026 (2)	0.030 (3)	0.023 (3)	0.0038 (19)	−0.0050 (17)	−0.001 (2)
C4	0.0295 (19)	0.028 (3)	0.021 (2)	−0.0039 (18)	0.0058 (16)	−0.001 (2)
C5	0.0207 (18)	0.027 (3)	0.032 (3)	0.0011 (19)	0.0009 (16)	0.000 (2)
C6	0.0229 (18)	0.023 (3)	0.025 (3)	0.0020 (17)	−0.0027 (16)	−0.001 (2)
C7	0.026 (2)	0.035 (3)	0.019 (3)	0.003 (2)	−0.0029 (17)	−0.004 (2)
Cl1	0.0208 (4)	0.0165 (5)	0.0182 (5)	0.0004 (4)	−0.0004 (3)	−0.0003 (4)
O1	0.0220 (14)	0.0215 (19)	0.046 (2)	0.0039 (12)	−0.0011 (14)	0.0018 (18)
O2	0.0350 (15)	0.0197 (18)	0.0240 (18)	−0.0006 (13)	−0.0028 (13)	0.0059 (15)
O3	0.0232 (14)	0.026 (2)	0.040 (2)	−0.0055 (13)	−0.0023 (13)	0.0019 (17)
O4	0.056 (2)	0.035 (2)	0.0192 (17)	0.0011 (18)	0.0047 (16)	−0.0051 (16)
N3	0.0189 (14)	0.0122 (19)	0.019 (2)	0.0020 (13)	0.0013 (13)	−0.0005 (15)
N4	0.0229 (17)	0.061 (4)	0.028 (2)	0.0016 (19)	0.0012 (16)	0.003 (2)
C8	0.0263 (17)	0.022 (2)	0.016 (2)	−0.0008 (19)	−0.0004 (15)	0.001 (2)
C9	0.0162 (16)	0.016 (2)	0.024 (2)	0.0016 (15)	0.0018 (15)	0.000 (2)
C10	0.0225 (18)	0.027 (3)	0.020 (2)	−0.0014 (17)	0.0054 (16)	−0.001 (2)
C11	0.0272 (19)	0.031 (3)	0.017 (2)	0.0028 (19)	−0.0008 (17)	−0.001 (2)
C12	0.0205 (17)	0.031 (3)	0.025 (3)	−0.0007 (17)	−0.0022 (17)	−0.006 (2)
C13	0.0180 (16)	0.019 (3)	0.027 (3)	−0.0033 (16)	0.0034 (15)	−0.004 (2)
C14	0.0213 (19)	0.033 (3)	0.024 (3)	0.0006 (18)	0.0052 (17)	0.000 (2)
Cl2	0.0207 (4)	0.0245 (6)	0.0188 (5)	−0.0032 (4)	0.0023 (3)	0.0007 (5)
O5	0.0409 (18)	0.032 (2)	0.026 (2)	−0.0075 (16)	−0.0042 (15)	0.0064 (17)
O6	0.0260 (17)	0.092 (4)	0.071 (3)	−0.0020 (19)	0.0140 (17)	0.046 (3)
O7	0.058 (2)	0.030 (2)	0.042 (2)	0.0098 (18)	−0.0241 (18)	−0.011 (2)
O8	0.058 (2)	0.021 (2)	0.043 (2)	0.0024 (17)	0.0123 (18)	−0.0073 (18)

### Geometric parameters (Å, º)

**Table:**

N1—C6	1.338 (5)	N3—C13	1.337 (5)
N1—C2	1.363 (6)	N3—C9	1.363 (5)
N1—C1	1.487 (5)	N3—C8	1.486 (6)
N2—C7	1.135 (6)	N4—C14	1.134 (5)
C1—H1A	0.9800	C8—H8A	0.9800
C1—H1B	0.9800	C8—H8B	0.9800
C1—H1C	0.9800	C8—H8C	0.9800
C2—C3	1.370 (6)	C9—C10	1.368 (6)
C2—C7	1.446 (6)	C9—C14	1.451 (5)
C3—C4	1.392 (6)	C10—C11	1.391 (5)
C3—H3	0.9500	C10—H10	0.9500
C4—C5	1.370 (7)	C11—C12	1.379 (7)
C4—H4	0.9500	C11—H11	0.9500
C5—C6	1.383 (7)	C12—C13	1.370 (7)
C5—H5	0.9500	C12—H12	0.9500
C6—H6	0.9500	C13—H13	0.9500
Cl1—O4	1.430 (3)	Cl2—O6	1.425 (4)
Cl1—O3	1.435 (3)	Cl2—O7	1.431 (4)
Cl1—O1	1.442 (3)	Cl2—O8	1.434 (4)
Cl1—O2	1.444 (3)	Cl2—O5	1.439 (4)
			
C6—N1—C2	120.0 (4)	C13—N3—C9	119.2 (4)
C6—N1—C1	120.2 (4)	C13—N3—C8	119.9 (4)
C2—N1—C1	119.8 (3)	C9—N3—C8	120.9 (3)
N1—C1—H1A	109.5	N3—C8—H8A	109.5
N1—C1—H1B	109.5	N3—C8—H8B	109.5
H1A—C1—H1B	109.5	H8A—C8—H8B	109.5
N1—C1—H1C	109.5	N3—C8—H8C	109.5
H1A—C1—H1C	109.5	H8A—C8—H8C	109.5
H1B—C1—H1C	109.5	H8B—C8—H8C	109.5
N1—C2—C3	121.2 (4)	N3—C9—C10	121.7 (3)
N1—C2—C7	116.7 (4)	N3—C9—C14	117.0 (4)
C3—C2—C7	122.1 (4)	C10—C9—C14	121.3 (4)
C2—C3—C4	118.8 (4)	C9—C10—C11	119.0 (4)
C2—C3—H3	120.6	C9—C10—H10	120.5
C4—C3—H3	120.6	C11—C10—H10	120.5
C5—C4—C3	119.6 (5)	C12—C11—C10	118.7 (5)
C5—C4—H4	120.2	C12—C11—H11	120.7
C3—C4—H4	120.2	C10—C11—H11	120.7
C4—C5—C6	119.6 (4)	C13—C12—C11	120.0 (4)
C4—C5—H5	120.2	C13—C12—H12	120.0
C6—C5—H5	120.2	C11—C12—H12	120.0
N1—C6—C5	120.8 (4)	N3—C13—C12	121.5 (4)
N1—C6—H6	119.6	N3—C13—H13	119.3
C5—C6—H6	119.6	C12—C13—H13	119.3
N2—C7—C2	178.7 (6)	N4—C14—C9	176.8 (5)
O4—Cl1—O3	109.8 (2)	O6—Cl2—O7	110.1 (3)
O4—Cl1—O1	109.3 (2)	O6—Cl2—O8	110.4 (3)
O3—Cl1—O1	109.2 (2)	O7—Cl2—O8	108.3 (2)
O4—Cl1—O2	110.3 (2)	O6—Cl2—O5	109.5 (2)
O3—Cl1—O2	109.3 (2)	O7—Cl2—O5	108.7 (3)
O1—Cl1—O2	109.0 (2)	O8—Cl2—O5	109.8 (2)
			
C6—N1—C2—C3	0.0 (7)	C13—N3—C9—C10	−0.3 (7)
C1—N1—C2—C3	−178.3 (5)	C8—N3—C9—C10	−177.4 (5)
C6—N1—C2—C7	178.6 (5)	C13—N3—C9—C14	179.9 (4)
C1—N1—C2—C7	0.3 (7)	C8—N3—C9—C14	2.8 (6)
N1—C2—C3—C4	0.1 (8)	N3—C9—C10—C11	0.4 (8)
C7—C2—C3—C4	−178.5 (5)	C14—C9—C10—C11	−179.8 (5)
C2—C3—C4—C5	0.1 (8)	C9—C10—C11—C12	−1.0 (8)
C3—C4—C5—C6	−0.2 (8)	C10—C11—C12—C13	1.5 (8)
C2—N1—C6—C5	−0.1 (7)	C9—N3—C13—C12	0.8 (7)
C1—N1—C6—C5	178.2 (5)	C8—N3—C13—C12	178.0 (5)
C4—C5—C6—N1	0.3 (8)	C11—C12—C13—N3	−1.4 (8)

### Hydrogen-bond geometry (Å, º)

**Table:**

*D*—H···*A*	*D*—H	H···*A*	*D*···*A*	*D*—H···*A*
C1—H1*A*···O1^i^	0.98	2.53	3.441 (5)	154
C1—H1*C*···O3	0.98	2.52	3.164 (5)	123
C3—H3···O5^ii^	0.95	2.54	3.326 (5)	140
C5—H5···O8^iii^	0.95	2.66	3.262 (6)	122
C6—H6···O1^i^	0.95	2.55	3.415 (6)	152
C6—H6···O4^i^	0.95	2.65	3.534 (6)	155
C8—H8*A*···O1^iv^	0.98	2.55	3.294 (6)	132
C8—H8*B*···O7^v^	0.98	2.57	3.538 (6)	169
C8—H8*C*···O6	0.98	2.51	3.425 (5)	156
C10—H10···O2^vi^	0.95	2.51	3.367 (5)	150
C12—H12···O2^vii^	0.95	2.52	3.347 (5)	145
C13—H13···O6	0.95	2.35	3.247 (6)	156

Symmetry codes: (i) *x*−1, *y*, *z*; (ii) −*x*+1, *y*−1/2, −*z*+2; (iii) −*x*, *y*−1/2, −*z*+2; (iv) *x*, *y*+1, *z*; (v) *x*+1, *y*, *z*; (vi) −*x*+2, *y*+1/2, −*z*+1; (vii) −*x*+1, *y*+1/2, −*z*+1.
