# Supplementary figures and images for: Crystal structure of 2-cyano-1-methyl­pyridinium perchlorate
Source: Acta Crystallogr E Crystallogr Commun. 2015 Oct 17;71(Pt 11):o852–3. doi: 10.1107/S2056989015019155 (PMC4645073; doi:10.1107/S2056989015019155)

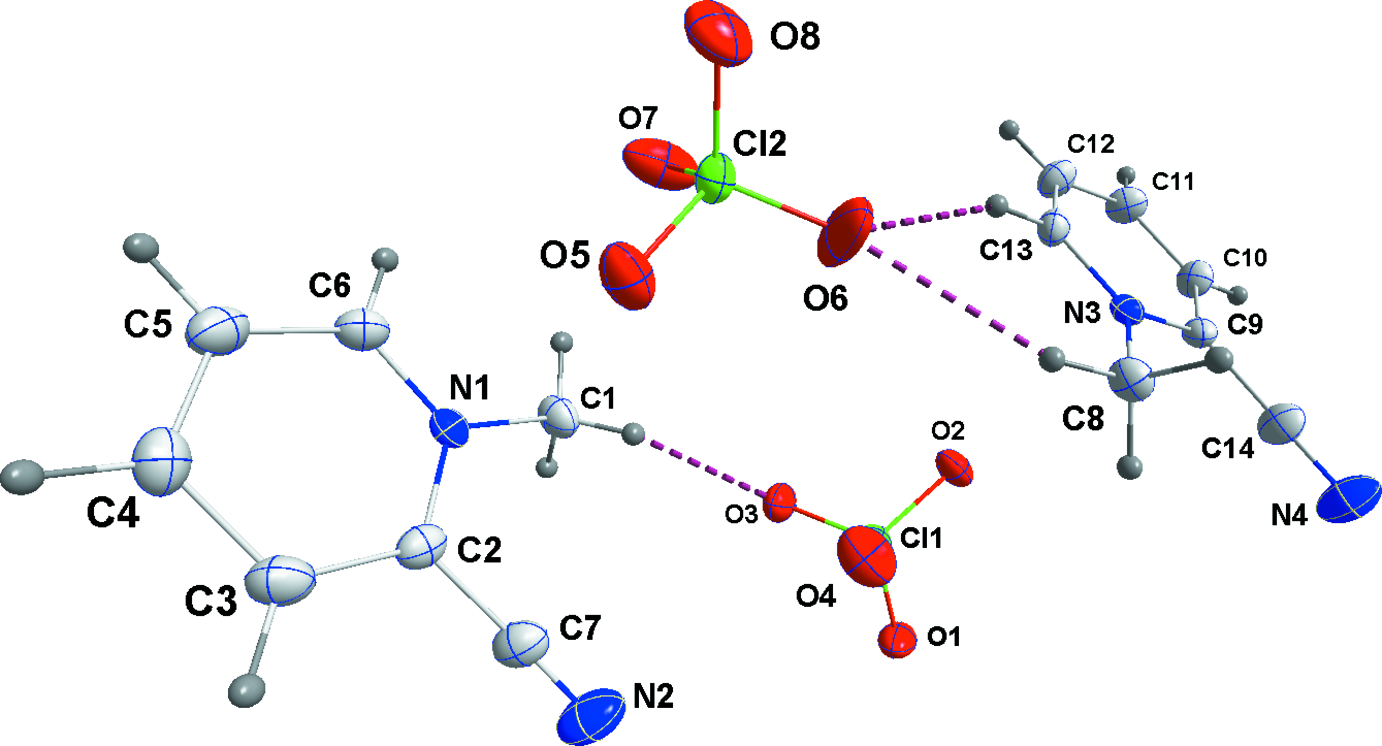

Supplement: Supplementary file 4 [file e-71-0o852-fig1.tif]

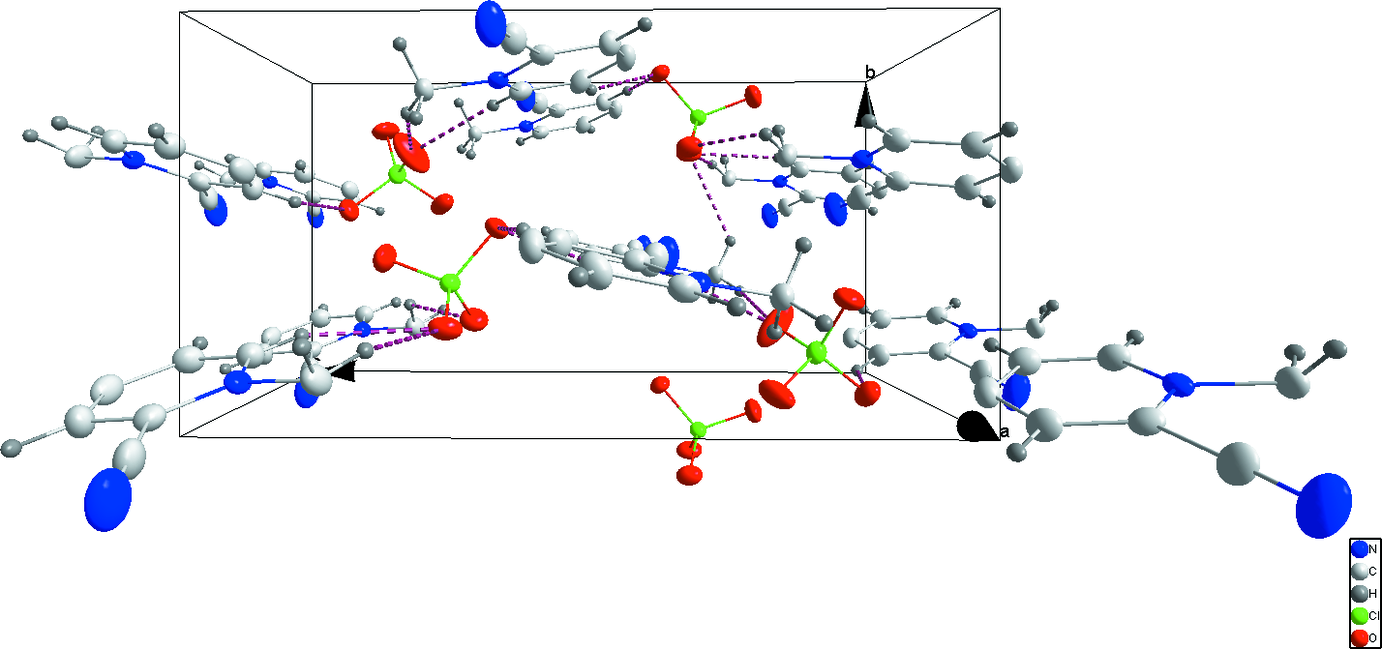

Supplement: Supplementary file 5 [file e-71-0o852-fig2.tif]

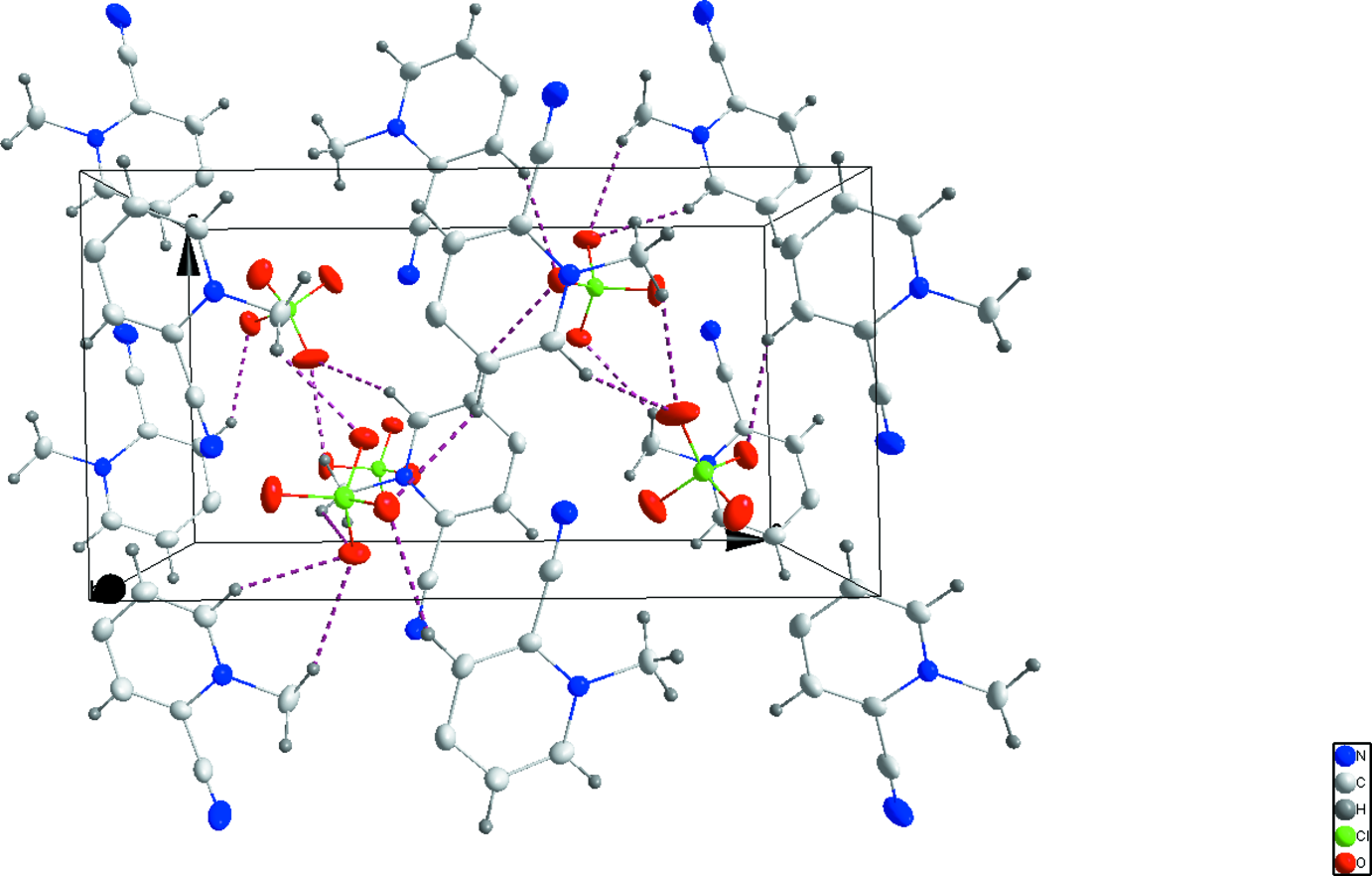

Supplement: Supplementary file 6 [file e-71-0o852-fig3.tif]
